# Voice disorders classification using machine learning: a scoping review

**DOI:** 10.3389/fdgth.2026.1800132

**Published:** 2026-06-08

**Authors:** Rijul Gupta, Craig T. Jin, Dhanshree R. Gunjawate, Duy Duong Nguyen, Brian Stasak, Antonia M. Chacon, Catherine Madill

**Affiliations:** 1 Computing and Audio Research Laboratory, School of Electrical and Information Engineering, The University of Sydney, Sydney, NSW, Australia; 2 Voice Research Laboratory, Sydney School of Health Sciences, Faculty of Medicine and Health, The University of Sydney, Sydney, NSW, Australia; 3 Department of Audiology and Speech Language Pathology, Kasturba Medical College Mangalore, Manipal Academy of Higher Education, Mangalore, India

**Keywords:** digital health, speech biomarkers, voice AI, voice disorder discrimination, voice disorder recognition

## Abstract

**Objectives:**

This review aims to identify the key barriers to clinical application of Machine Learning (ML) in multi-class voice disorder classification.

**Design:**

Scoping Review.

**Methods:**

A comprehensive scoping review of research published between 2013 and May 2025 in seven clinical and engineering databases was conducted. Articles that applied ML techniques to classify voice disorders were examined, excluding publications limited to binary classification (e.g., healthy vs. pathological). Data were extracted from the included articles to analyze patterns in the specific voice disorder classification classes, database selection, the input data attributes, vocal tasks, diagnostic labelling, and the applied ML classification techniques.

**Results:**

In total, 10,401 articles that addressed voice disorder classification were screened from which 80 used ML techniques for multi-class classification. Results revealed considerable variation in selection of databases, voice disorder diagnostic labels, amount and type of input data (e.g., voice tasks and demographics questionnaire), and classification techniques. These inconsistencies prevent robust comparisons and therefore identification of state-of-the-art solutions, which would typically mature to clinical applications.

**Discussions:**

Variations in classification tasks make it difficult to compare results across studies. The inconsistency found in terms of class imbalance, sample size, and total number of classes investigated, means there is no baseline for comparing and exploring various classification techniques. Finally, variations in testing methods such as using different test set types and sizes or using cross validation limit comparisons across articles.

**Conclusions:**

This review identified considerable variations in the diagnostic labels associated with voice disorder classification, data availability per selected label, and testing methodology. Such variation limits comparability and undermines the generalization of ML models. The lack of consensus across the automated classification pipeline – from selection of which disorders should be classified using ML systems, to constructing test sets and measuring performance – are likely to be critical barriers to clinical application. These barriers must be addressed to realise the potential for using voice as a biomarker of other systemic diseases.

## 1 Introduction

Voice disorder classification is a crucial early stage in the increasingly expanding field of Voice AI (artificial intelligence), which is expected to revolutionize healthcare (1). As the potential for using voice as a biomarker of voice-affecting conditions grows—from monitoring neurological and cardiovascular health to detecting degenerative diseases —its role in voice disorder classification becomes a critical testing ground. This is because AI is a non-invasive and objective method that can extract voice features, determine whether a voice is normal or disordered, and classify the severity of disorder (2). Voice AI is expected to detect diseases that may subtly influence the voice as its algorithms work on acoustic measures reflecting vocal characteristics. Acoustic voice recordings are important data sources to reliably detect and diagnose voice disorders using AI, with broader applications as the health system faces significant challenges related to specialist shortage, long wait time, and diagnosis reliability.

Despite a significant increase in the number of studies during the last decade, no study has successfully bridged the gap between experimental research and clinical application. The Vox4Health (3) has reached clinical trials in 2018 but failed to transition into an accessible clinical product. It remains unclear whether this failure is due to limited clinical applicability, inconsistent ML results, or other implementation challenges (e.g., limited data resource, diagnostic metadata agreement). Identifying these barriers is crucial to guiding future research and ensuring the transition to clinical use.

The challenge of voice disorder classification from acoustic signals is to reliably and accurately support and even replicate the decision making of clinicians. Ear-Nose-Throat specialists (ENT) and speech-language pathologists (SLP) use several different “diagnostic classification frameworks”^1^ for voice disorders such as the Classification Manual for Voice Disorders-I (4), Morrison et al. (5), Baker et al. (6), and Payten et al. (7). These frameworks are structured differently with some being based on symptoms, while others are based on etiology. All frameworks have multi-level classification, involving grouping of different diagnoses into classes of similar symptoms or causes. For example, Payten's hierarchical framework describes 3 *types* of voice disorders, Organic, Muscle Tension and Psychogenic, under which there are *sub-classes* (e.g., Primary, Secondary and Adaptive Muscle Tension) followed by a list of labels for different individual *diagnoses*. The problem is that ENTs and SLPs do not always use the same frameworks, leading to a lack of consensus of terminology and diagnostic classification within the field. Despite this, the interest in automated classification of voice disorders remains high.

**Figure 1:**
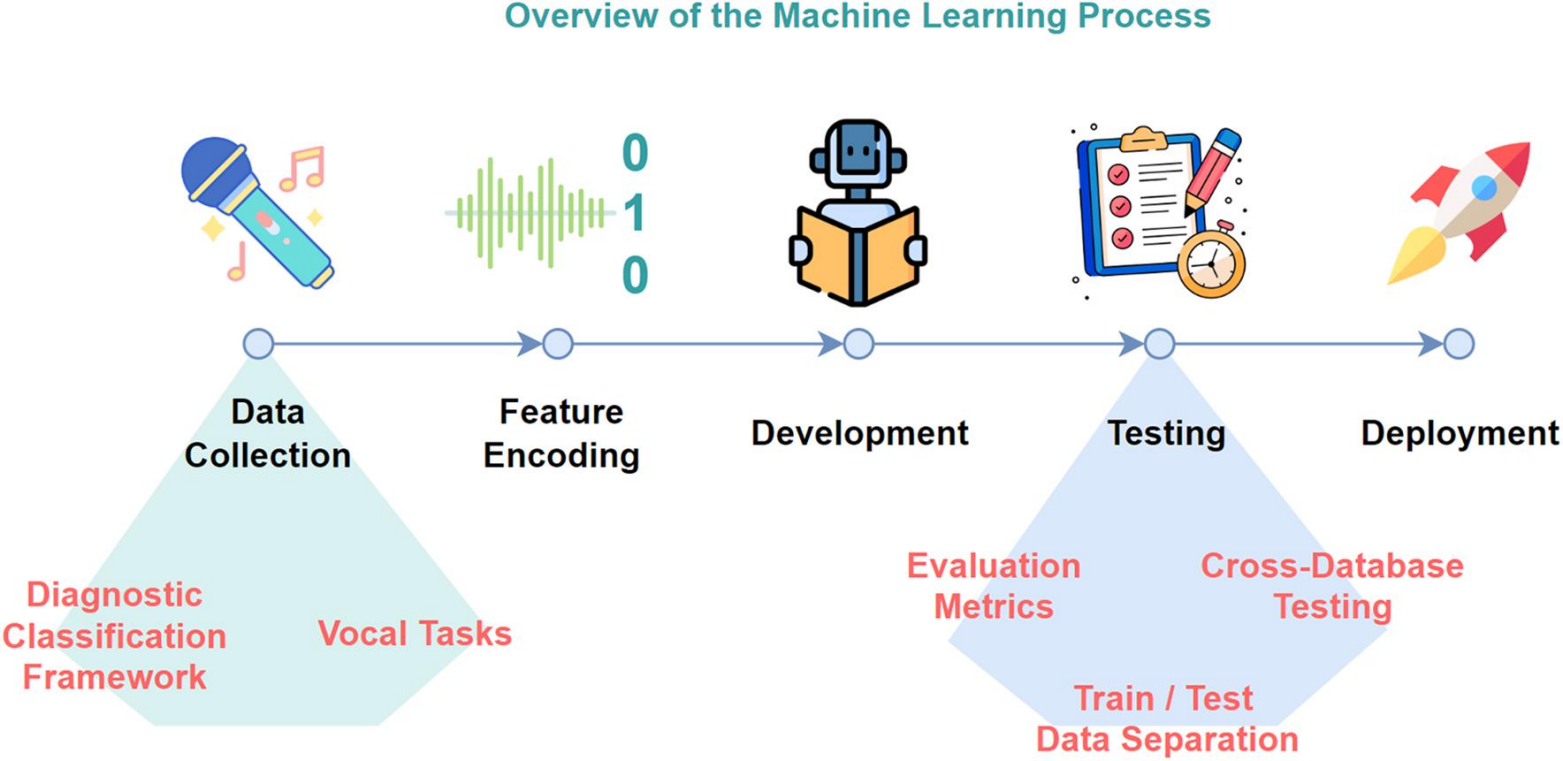
Overview of the machine learning pipeline for voice disorder classification, from data collection to clinical deployment. Sub-steps where current research lacks rigor and where critical improvements are needed are highlighted in red. Icons from Flaticon: “Karaoke” by Flat Icons, “Sound Waves” by Freepik, “Learning” by Freepik, “Exam Time” by brick-stock and “Project Launch” by Freepik, licensed under Flaticon License.

Figure 1 outlines the five stages of an ML project: 1) **Data Collection** involves identifying disorders to classify and gathering relevant data. 2) **Feature Encoding** transforms raw data into numerical features, including spectrogram generation from audio or mapping categories to numbers. 3) **Development** selects, develops and trains ML models on the data. 4) **Testing** evaluates ML models using unseen data and eventually clinical trials. 5) **Deployment** enables real-world applications.

The majority of research in voice disorder classification has designed the Data Collection stage for exploring binary classification e.g., between healthy and disordered voice samples, and between healthy and a specific disorder [e.g., Glottic Neoplasm, Recurrent Laryngeal Nerve Palsy, Spasmodic Dysphonia, Vocal Fold Paralysis (8–10)]. This approach may not be clinically useful as the outcome only allows discrimination between disordered and non-disordered status or only applies to several voice disorders. To date, nine literature reviews have been published, none of which have provided consistent data about the application of multi-class ML classification. In the most recent review, Liu et al. (11) examined the clinical readiness of ML systems in voice disorder classification. They found that only 24 of the selected studies (29%) focused on classification, within which an unspecified subset addressed multi-class classification. Al-Hussain et al. (12) provided valuable insights by focusing on articles published before 2021 that compared a particular disorder with healthy control subjects, though it is limited to binary classification. Hecker et al. (13) examined trends in the literature before 2021, offering a broad overview but without a direct comparison of methodologies or a focus on challenges to clinical application. Idrisoglu et al. (2) explored articles published before 2023, addressing voice disorders in neurological diseases like Parkinson's and dementia, which offered useful context but did not fully capture the breadth of voice disorders. Syed et al. (14) focus on studies utilizing published databases such as the Saarbruecken Voice Database (SVD) (15), Massachusetts Eye and Ear Infirmary (MEEI) (16), and Arabic Voice Pathology Database (AVPD) (17) which, while comprehensive, excluded research that use unpublished or private data, limiting their scope. Sindhu and Sainin (18) examined studies using Deep Learning (DL) techniques, which, although promising, represented a smaller subset of methods in voice disorder classification. A two-part scoping review (19, 20) investigated variations in terminology across the literature, helping to clarify definitional differences but not focusing on methodological or clinical gaps. Finally, five non-systematic reviews provide broad overviews of the field: a survey of ML techniques and features (21), a focused review on Mel Frequency Cepstral Coefficients (MFCC) (22), and three reviews covering subset of datasets, features, and models used in the voice disorder classification pipeline (23–25). These reviews offered broad insights but lacked a deep exploration of specific challenges in clinical application.

While distinguishing between healthy and pathological voice samples is valuable, clinicians require ML tools that can process beyond simple differentiation to assist them accurately identify specific pathologies. The real benefit lies in a tool's ability to classify voice samples into specific subtypes of pathology, facilitating timely and efficient redirection of patients to the appropriate specialists, ultimately reducing the burden on the primary healthcare system. Researchers are beginning to recognize the limitations of binary classification between healthy and pathological only as evidenced by emerging research on multi-class classification (26–28) which distinguishes between multiple pathologies, or multiple pathologies and healthy samples.

This review aims to identify key challenges and methodological issues in the existing literature on multi-class voice disorder classification, with a focus on factors that may impact the efficacy and clinical implementation of ML systems in this domain.

## 2 Methods

To systematically navigate the collected 24,696 research articles, the Preferred Reporting Items for Systematic Reviews and Meta-Analyses extension for Scoping Review guidelines (PRISMA-ScR) (29) were used. The PRISMA-ScR checklist for this review has been provided in Supplementary Appendix A. The scope of the review was restricted to studies incorporating multi-class classification.

### 2.1 Protocol

The protocol for this review was published in BMJ Open on 24 February 2024 (30).

### 2.2 Data sources

MEDLINE, Embase, Scopus, CINAHL, MEDLINE, Embase, Scopus, CINAHL, Compendex, Web of Science and IEEE Xplore were selected for literature searching for the review. This represents a coterie of databases that encompass both the medical and engineering discipline which are critical to address the inter-disciplinary nature of this work. We included both conferences and journals articles. Grey literature was not included.

### 2.3 Search strategy

The search strategy included six different search areas (Voice disorders, Organic voice disorders, Muscle tension dysphonia, Functional voice disorders, Engineering challenge, Engineering technique) that were gathered from keywords within existing literature and suggestions from the authors, which is available in Supplementary Appendix B. These keywords were then combined to formulate a comprehensive search strategy with the assistance of librarians at The University of Sydney Library, NSW, Australia. The search strategy was then refined to accommodate the syntactical requirements for different databases. The full search strategy is provided in the Supplementary material Appendix C.

Following the explosive growth in ML research since the origins of AlexNet (31) in 2012, we conducted a search from 2013 to 2024. Since broad search terms were used such as “classification” instead of specific techniques like “machine learning” or “neural networks”, our search resulted in a collection of related literature substantially larger than would otherwise be obtained, and three times larger than the largest existing scoping review on this topic (19). Repeat searches in 2024 and 2025 were conducted to capture any literature published between 2023 and May 2025. The final search was completed on 19th of May 2025.

### 2.4 Article selection process

The search results from the afore-mentioned data sources were imported into a web-based literature review management tool, Covidence (32), which was used for both de-duplication of results and Title and Abstract screening. The selection criteria were discussed with all reviewers as well as uploaded into Covidence for easy access during the screening process.

#### 2.4.1 Inclusion criteria

Publications were selected if they were peer-reviewed journal articles and conference proceedings that met the following criteria:

1. Investigated diagnostic classification of voice disorders using ML systems (in all or part of the study)
2. Explored the application of ML for screening, identification and assessment of voice disorders
3. Included individuals for ML investigation with and without voice disorders irrespective of their language, region, gender, age, and ethnicity
4. Were published in the English Language
5. Were published from 1st January 2013 to 19th May 2025

#### 2.4.2 Exclusion criteria

Publications were excluded based on the following criteria:

1. Describes visual-only inspection e.g., laryngeal imaging, laryngoscopy findings
2. Describes binary-only classification
  - Healthy vs. Pathological
  - Healthy vs. Any single disorder (e.g., Parkinson’s disease)
  - One disorder vs. another disorder
  - One disorder vs. collection of other disorders (for e.g., Parkinson vs. all voice disorders in a dataset)
3. Focus solely on pediatric disorder detection because there are no public databases that have pediatric samples
4. Investigates only app development
5. Were published in languages other than English
6. Consists only of editorials, ongoing studies, or working studies

#### 2.4.3 Title and abstract screening

All reviewers had been working in their respective fields for approximately ten years. The Title and Abstract screening was performed by two reviewers. The first author screened all the articles, while the screening task was divided among the other authors based on their availability. In this stage, papers that did not include ML techniques were excluded.

#### 2.4.4 Full text screening

A single reviewer with engineering expertise (the first author) screened all the articles resulting from the Title and Abstract screening stage. The articles excluded at this stage were discussed with other reviewers to ensure that the reason for exclusion was valid. Random samples of excluded articles (20%) were discussed with other reviewers to verify adherence to the eligibility criteria.

#### 2.4.5 Data extraction

A data extraction tool was implemented in Microsoft Excel, and captured databases used, participant demographics, diagnostic methods, classification labels, model details, training/testing splits, evaluation metrics, and cross-study comparisons, including baseline models, interpretability, and hardware requirements. The full data extract is available online on our github repository^2^.

Data extraction was independently performed by two reviewers, wherein one reviewer extracted the data relating to the databases and another extracted the remaining content of interest. Several analysis worksheets were constructed from the extracted data to organize results.

#### 2.4.6 Synthesizing results

Multiple MS excel worksheets were created from the main data extraction worksheet to track variations in all elements of the ML process shown in Figure 1 as well as to generate summary statistics. Python scripts were used to study relationships between different classification labels and plot graphs. These graphs enabled reviewers to determine which diagnosis were compared against each other and assess data availability for any given diagnosis across different research articles.

## 3 Results

**Figure 2:**
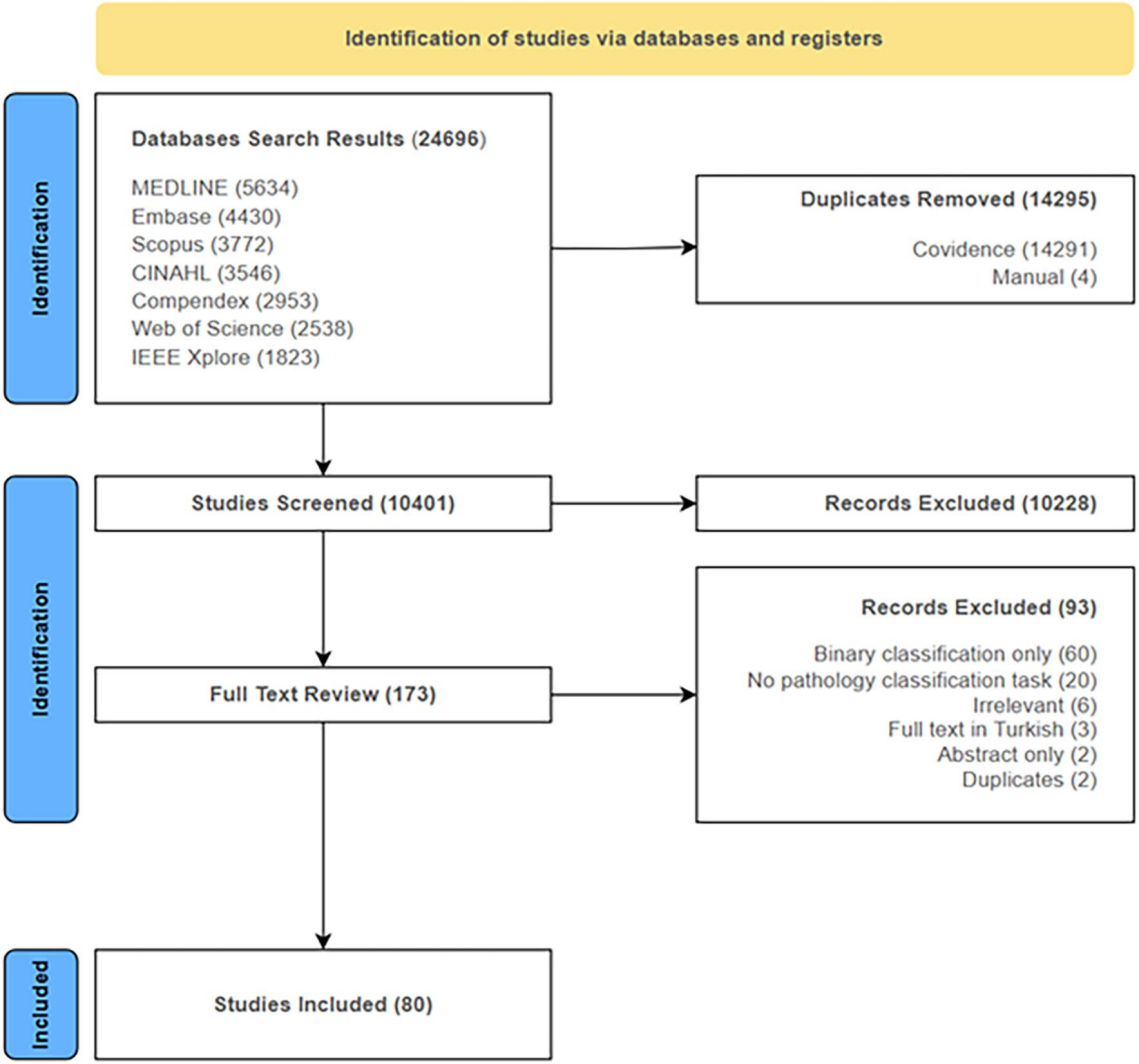
PRISMA flowchart outlining the serial review workflow.

As presented in Figure 2, the literature search produced a total of 24,696 results. After de-duplication 10,401 articles underwent Title-and-Abstract screening using the above-mentioned inclusion and exclusion criteria. Eight reviewers – including three engineers, one otolaryngologist and four speech pathologists – screened the articles. 173 articles remained after the Title-and-Abstract screening and were used for full-text review.

Sixty of the articles excluded in the full-text review focused on binary classification, which was not apparent during Title-and-Abstract screening. Although these articles used different voice pathologies from their selected databases, they subsequently combined the different pathologies to compare against a single class in a one-vs.-all binary classification task – such as Healthy vs. Dysphonia, Parkinson's, Laryngitis, and Heart Failure (33). This process occurred in few articles, which were excluded at the data extraction stage upon identifying that they did not perform multi-class classification when we attempted to extract relevant data. For instance, Geng et al. (34) provided a confusion matrix for multi-class classification, but the article focused solely on binary classification tasks and did not explain the confusion matrix.

Finally, 80 articles were included for data extraction and analysis. The results of the data extraction are outlined in the following subsections, while the full extracted data is available online on our github repository.

### 3.1 Databases

15 public and 23 private databases were used across the 80 articles. The databases were either publicly available or unpublished, where unpublished databases are defined as data collected directly by the authors or by a participating organization, such as a university clinic. Unpublished databases are neither made available to other researchers freely nor advertised as available through a formal request process.

Most articles used a single database (*n* = 68). The distribution of multiple databases used across the remaining 11 articles is: two databases (*n* = 7), three databases (*n* = 2), and four databases (*n* = 3). The Saarbruecken Voice Database (SVD) was used by 28 articles, private databases were used by 23, MEEI by 17 publications. Far Eastern Memorial Hospital (FEMH) 2018 appeared in 9 articles and other databases were referenced by fewer than 5 articles. Two articles did not specify which databases they used (Table 1).

**Table 1:** Database usage across the articles in this review. Naming convention of databases follow articles included in this review.

Database	Publications
SVD (15)	28
Unnamed private database	23
MEEI (16)	17
FEMH 2018	9
Voice ICar fEDerico II (VOICED) (75)	4
FEMH (private)	3
FEMH 2019	2
PC-GITA (76)	2
CLP-GPRS (77)	2
Pr ´ıncipe de Asturias (PdA) (78)	2
AVPD (17)	2
CIEMPIESS (79)	1
Hospital Universitario Prłncipe de Asturias (HUPA) (80)	1
LURCO-Lab DPV (81)	1
Unnamed public Parkinson's DB (82)	1
Unspecified	2

The FEMH 2018 and FEMH 2019 challenge datasets were made available to participants during the respective challenges but are no longer publicly accessible. Additionally, researchers affiliated with FEMH continue to publish articles using data originating from the hospital. However, it is unclear how much of this data overlaps with the challenge datasets or how much overlap exists between the FEMH 2018 and FEMH 2019 challenge datasets.

### 3.2 Classification labels

**Figure 3:**
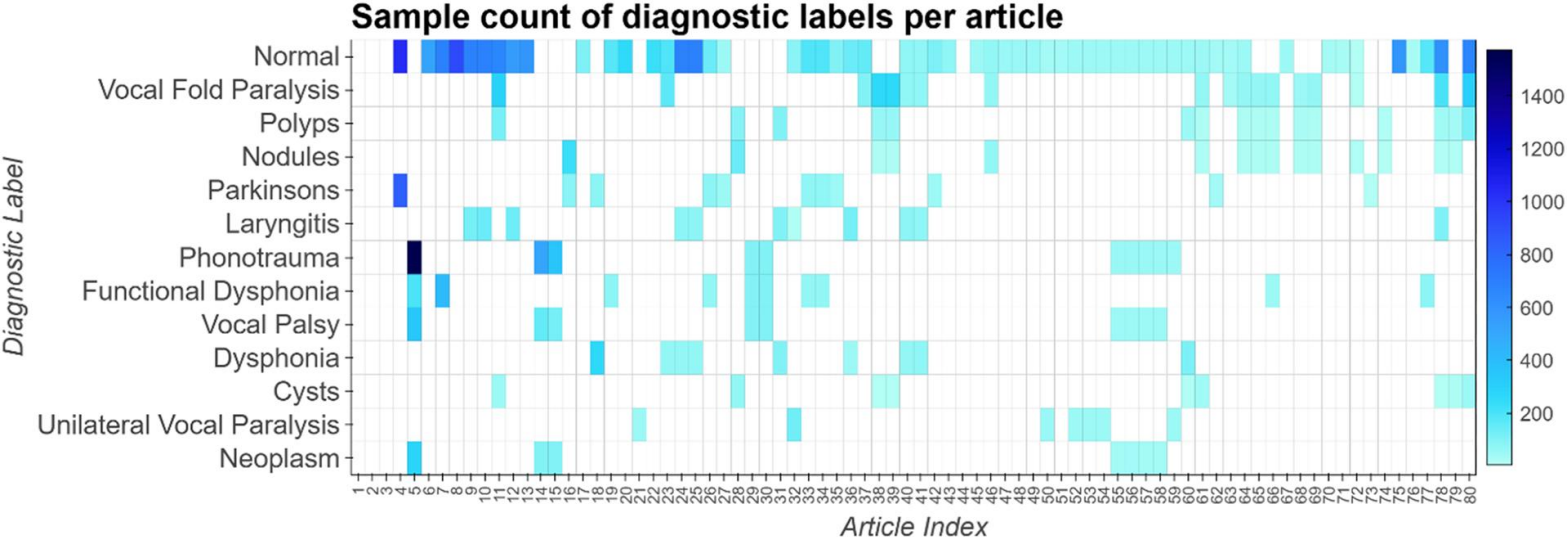
Diagnostic labels across the included articles sorted by frequency of use across articles where a label is used by at least 5 articles. Darker color saturation indicates higher number of samples reported in the article. Scanning across any column reveals combination of labels used in an article.

Laryngeal conditions considered for classification tasks were highly varied across the literature as shown in Figure 3 and included combinations of *types*, *sub-classes* and *individual diagnosis*. Of the 80 reviewed articles, 63 included a control group, while 17 did not. A total of 73 distinct classification labels were identified, although one article (35) inconsistently reported classification labels, making it unclear which labels were used for experiments.

Most articles reviewed classified between 4-classes (35 articles) or 3-classes (30 articles), with fewer studies (14 articles) comparing more than 4 classes. Disorder types were usually selected based on data availability, and disorders with low data availability were usually excluded. We provide a few examples of the specific voice disorder types for the 4-class articles:

- Neoplasm, Normal, Phonotrauma, Vocal Palsy [5 articles (26–28, 36, 37)]
- Dysphonia, Laryngitis, Normal, Vocal Fold Paralysis [3 articles (38–40)]
- Laryngitis, Normal, Unilateral Vocal Paralysis, combined (Vocal Nodules, Polyps, Cysts) (3 articles (41–43)

The specific voice disorder types for the 3-class articles include:

- Nodules, Polyps, Paralysis [4 articles (44–47)]
- Neoplasm, Palsy, Phonotrauma [3 articles (26, 48, 49)]

None of the studies referred to the use of a published diagnostic classification framework for voice disorders in the labelling of the disorders. To the best of our knowledge no studies and/or databases reported or described a systematic or consensus approach to labelling or reported confidence in diagnosis data [as recommended by Eastwood et al, 2015 (50)]. Only one (51) of the 80 articles reported a consensus-based methodology in their study. Even this was only used for severity, and not for diagnosis.

### 3.3 Heterogeneity and availability of data

**Figure 4:**
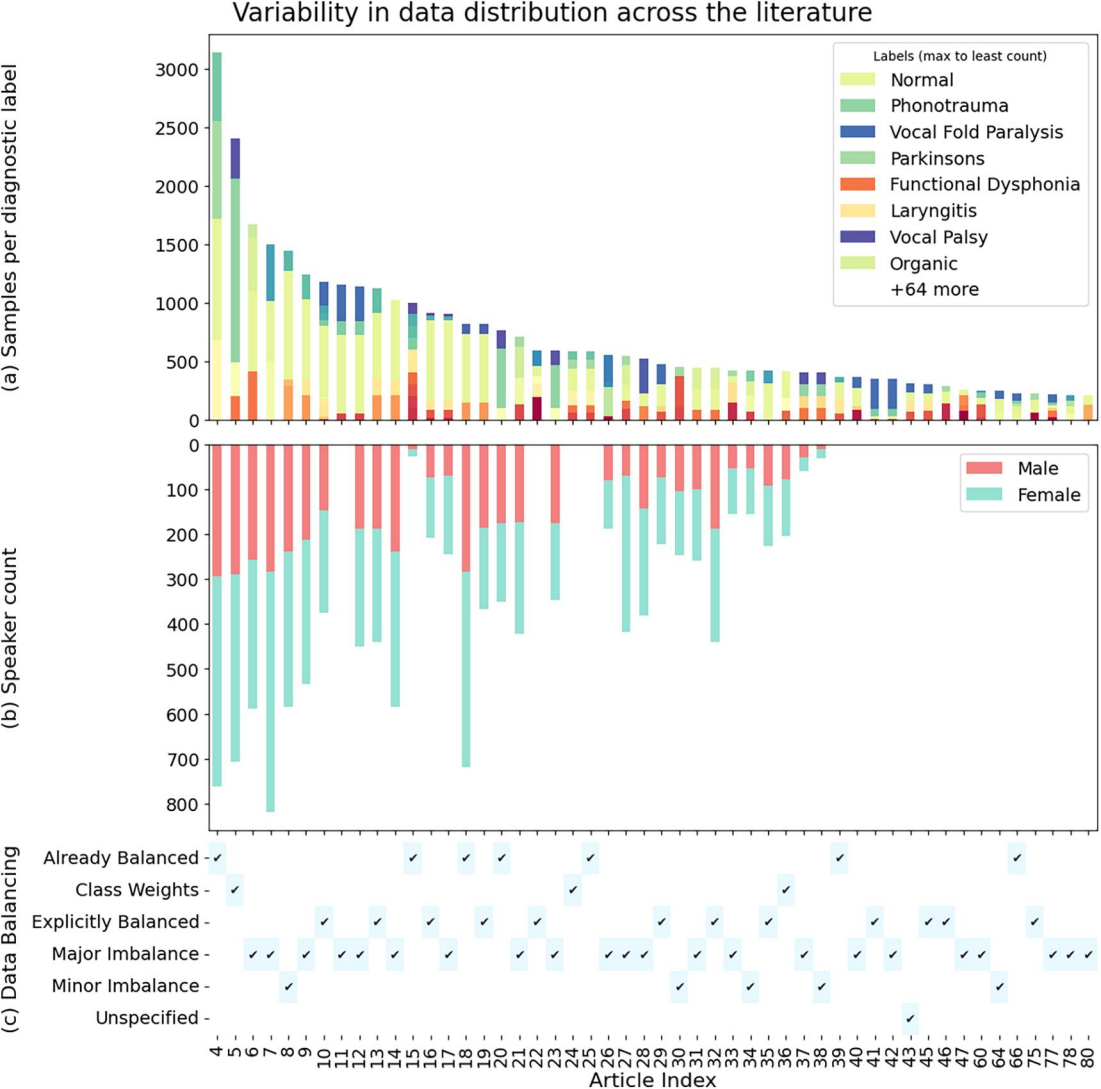
Top 50 articles based on total reported data samples used in experiments. **(a)** Distribution of samples per diagnostic label (More colors in a bar represent more diagnostic labels). **(b)** Reported speaker demographics, several papers did not report demographics and are therefore absent in the figure. **(c)** Data balancing strategy for training data where reported.

 Figure 4 illustrates the variations in the data used for experiments across the literature through a sampling of the top 50 articles. The articles were ranked based on the total number samples used within the experiments. Three articles did not report quantitative measures of data. Seventy-three different diagnostic labels were used across this set of 50 articles, within which normal samples outnumbered every pathological sample as shown in Figure 4(a). Forty-five articles (58.1%) did not provide demographic information for their data. Thirty out of the 35articles that reported demographic information had more female than male participants as evidenced in Figure 4(b) which is consistent with research documenting a higher incidence of voice disorders in women than men (52).

Figure 4(c) shows that imbalanced datasets were used by the majority of articles, and 41(50%) articles had a major imbalance in which the sample counts for the diagnostic labels with the greatest and fewest numbers of data samples differed by more than 20%. Sixteen articles explicitly balanced the data by reducing samples of larger classes, creating synthetic data with random noise sampling, or oversampling smaller classes. Nine articles had only small differences in data available for each diagnosis, while in three articles researchers adjusted the model settings to pay more attention to underrepresented diagnoses so that those categories were not ignored during training. Eight articles used data that was balanced in terms of input audios and/or speakers, and three articles did not provide sufficient details of data balance.

Across the reviewed literature, there were 19 instances where pairs of classes showed varying sample distributions between studies. In some articles, one class had more samples than the other, while in other studies, the distribution was reversed for the same classes. For instance, in (53) Hyperkinetic dysphonia had 72 samples, while normal had 55; in (54) with Hyperkinetic dysphonia had 283 samples, and normal had 927.

### 3.4 Classification pipeline

**Figure 5:**
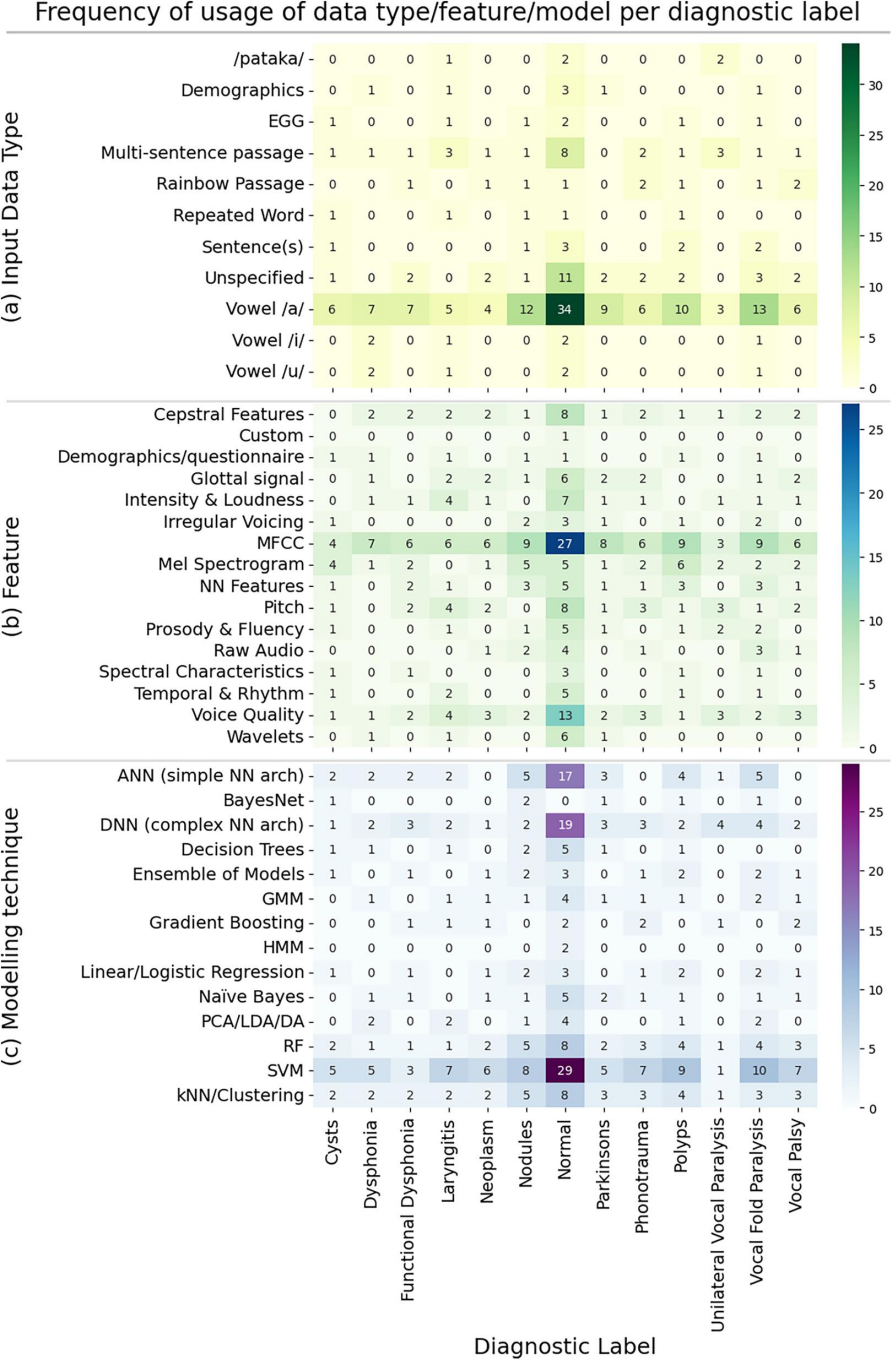
Frequency of use of **(a)** input data, **(b)** feature types, and **(c)** modelling technique for classification of diagnostic labels used in at least 5 articles. Scanning across a row and column indicates how often a data type, feature or model was used across the literature and diagnostic label.

Various combinations of data inputs, feature representations (both raw and processed), and modeling techniques have been explored to classify distinct diagnostic labels (refer to Figure 5). As can be seen, despite these variations, most studies have utilized the following structural combination: the vowel/*a*/ as the vocal task, MFCC as the feature, and Support Vector Machines (SVM) as the model.

#### 3.4.1 Vocal tasks and input data

Vocal tasks and input data for ML processing varied across articles, including sustained vowels, word repetitions, reading passages [e.g., the Rainbow Passage (55)], demographics, and electroglottography (EGG). As shown in Figure 5(a), the most frequently used vocal task was the vowel/*a*/, followed by multi-sentence passages such as short tales or scripts. Few studies incorporated non-vocal inputs such as EGG or demographic/symptomatic questionnaires. Fifty-three papers used a single type of input data, 11 used two types, and 3 incorporated three different types. Additionally, ten articles did not clearly specify which tasks they used [e.g., (54, 56, 57)].

While most studies relied on only one category of input data (e.g., a single speech task or demographic feature), a few incorporated multiple data sources. Among the three studies that used three types of inputs, one combined sustained vowel/*a*/, demographic information, and EGG signals, while the other two included multiple vowel sounds but the same vocal task type (/*a*/,/*i*/, and/*u*/). The nine studies that used two categories of input data explored diverse combinations, including vowel sounds with demographic data, structured reading tasks, or EGG.

Seven studies (26, 42, 46, 53, 58–60) addressed recording conditions through various techniques, including denoising systems, voice activity detection (VAD), and manual noise removal. Two studies (42, 58) trimmed silence from recordings, one study (59) relied on expert verification to ensure audio quality, two studies (26, 46) used preprocessing steps to enhance feature extraction in 2 studies, and 2 studies (53, 60) created noisy samples to improve model adaptability to real-world conditions.

#### 3.4.2 Classification features

The features used across articles were categorised into several groups: Raw Audio, Neural Network (NN) features [e.g., Wav2Vec (61)], Mel Spectrogram, MFCC, Wavelet Transform-based features, Glottal Signal Flow-based features, Demographics and Questionnaires, Custom (bespoke algorithms), Pitch-related features (e.g., F0), Spectral Characteristics (e.g., Spectral Energy), Voice Quality features (e.g., Shimmer and Jitter), Intensity features (e.g., amplitude), Temporal & Rhythm-based features (e.g., ratio of silence), Irregular Voicing features (e.g., Irregular Voicing Probability Threshold), and Prosody & Fluency-based features (e.g., Signal Stats). A detailed breakdown of each category is available in the Extraction instrument under the worksheets “Features” and “Grouping Other Features”.

Figure 5b illustrates the predominance of MFCC in most studies (*n* = 43), followed by voice quality features (*n* = 19). In contrast, only 8 studies have explored specialized features such as NN features or wavelet techniques, and only 3 articles used demographic data.

Demographic or symptomatic data is likely encoded using label encoding, the standard approach for ML models. This was either explicitly stated by the authors or inferred from the absence of any reference to alternative methods.

#### 3.4.3 Classification systems

Classification systems were divided into 14 different categories: complex Deep Neural Network architectures that are constructed from different types of neural networks (DNN), simple feedforward neural network architectures (ANN), Support Vector Machines (SVM), Hidden Markov Models (HMM), Gaussian Mixture Models (GMM), k Nearest Neighbors (kNN)/Clustering, Random Forests (RF), Decision Trees, Gradient Boosting, Linear/Logistic Regression, Naïve Bayes, BayesNet, Voting Classifier, and Principal Component Analysis/Linear Discriminant Analysis/Discriminant Analysis.

Figure 5(c) highlights the predominant use of SVMs, followed by DNNs and ANNs, while other ML approaches appear less commonly. Additionally, 25 articles apply dimensionality reduction for feature selection before classification, and four articles use ensembles of models. Nine articles applied transfer learning, self-supervised pre-training, or audio foundation models such as Whisper, wav2vec, AudioSet-pretrained networks, or Vision Transformers, almost exclusively in 2021 or later.

Although some articles examined ML model fit, none of the 80 articles examine model interpretability, either in terms of neural network focus or feature importance in predictions.

#### 3.4.4 Training/testing strategy

Out of the 80 articles, 52 used cross-validation (ranging from 4-fold to 10-fold and leave-one-out cross-validation), 25 articles used a portion of the total data for testing and three articles did not specify how they split the data for training and testing (62–64). Cross-database testing was conducted in only one study (65). With test sets averaging around 100 samples (118.19 ± 228.79) and 47 articles using four or more classes, they effectively had 25 or fewer samples per class.

Given that test sets averaged around 100 samples (117.64 ± 161.23), and 49 articles involved classification into four or more classes, this implies that these studies typically had 25 or fewer samples per class — raising concerns about the statistical robustness of their evaluations.

### 3.5 Metrics

**Figure 6:**
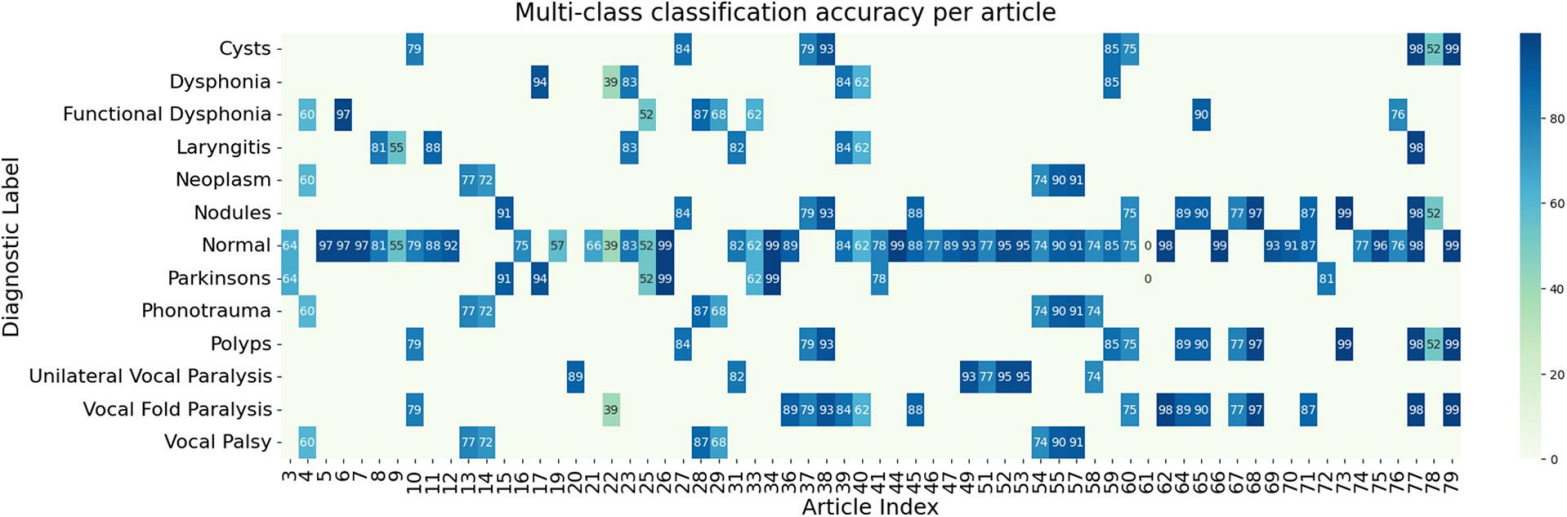
Reported multi-class classification accuracy for articles that contain diagnostic labels used by at least 5 articles.

Although most articles reported accuracy in weighted, total, or balanced formats, others prioritized alternative metrics like Unweighted Average Recall (UAR), True Positive Rate (TPR), or False Positive Rate (FPR). Among articles that include diagnostic labels appearing in at least five studies, reported accuracy ranges from 39% to 99% as shown in Figure 6.

It should be noted that these values do not directly represent the performance on any single diagnostic label, as results are derived from varying subsets of labels and influenced by different averaging methods. This extreme variability reflects inconsistencies across all preceding stages—differences in dataset composition, feature selection, and modeling choices—making direct comparisons between studies impractical.

## 4 Discussion

We conducted a comprehensive review of the past decade's literature to identify key challenges hindering the transition of automatic voice disorder classification systems from research to clinical application. In contrast with (2, 11, 12) we identified 80 articles specifically addressing multi-class classification—a crucial step towards clinical adoption. While data inconsistencies are widely recognized as a barrier to implementation, we provide a detailed breakdown — as outlined in Figure 1 — of how these inconsistencies arise throughout the ML pipeline. This includes variation in database selection, class definitions, data usage per class, application of data balancing techniques, and the reporting and comparison of results. In this section we highlight the key challenges where methodological improvements are needed to enhance reproducibility, validity, and ultimately, clinical impact.

### 4.1 Data collection

Many widely used voice disorder databases such as SVD and MEEI were not originally designed for ML applications. They were compiled from clinical records without standardized diagnostic protocols, often lacking consistent metadata, gold-standard diagnostic confirmation (e.g., laryngoscopy), or inter-rater agreement. Additionally, these datasets exhibit substantial class imbalance: some disorders have only sparse examples, making evaluation metrics highly sensitive to individual samples and limiting the generalizability of trained models.

In response to these limitations, newer datasets such as Voiced, AVPD, and Bridge2AI (66) have been developed with ML as a primary use case. These datasets emphasize diagnostic consistency—typically requiring laryngoscopic confirmation and consensus from multiple clinicians—and aim to improve class balance by prioritizing the collection of data from underrepresented voice disorders. Bridge2AI, in particular, explicitly addresses small class sizes to ensure better evaluation stability and broader clinical relevance.

However, even these improved datasets do not report the diagnostic classification frameworks used, and most research continues to rely solely on the/*a*/ vowel task despite the availability of more diverse vocal tasks.

#### 4.1.1 Diagnostic classification framework

A key limitation in the current research is the lack of consistent reporting on the diagnostic classification frameworks used for labelling or grouping pathologies. Only one study, da Silva Moura et al. (67) cited a classification framework (68). This omission hinders the ability to replicate findings, compare studies effectively, and aggregate datasets in a meaningful way.

We recommend that future studies explicitly state the classification framework used for labelling data. Frameworks based on etiology should be also reconsidered in this context, as ML models focus on symptoms (e.g., voice variations) that may or may not be directly linked to the underlying causes of a disorder and different etiologies can result in similar vocal symptoms.

Beyond inconsistent reporting, there is a deeper mismatch between clinical diagnostic frameworks and the way ML class labels are typically constructed in the reviewed literature. Clinical frameworks (6, 7) are hierarchical and grounded in etiology or symptomatology, whereas the ML class lists in the reviewed articles are flat and constructed pragmatically from whatever labels were available in a given database. Diagnoses in clinical practice are usually confirmed laryngoscopically and often by inter-rater agreement, while database labels are often of unknown provenance and were rarely re-validated for the ML task.

This mismatch is consequential for clinical translation. A model that distinguishes specific diagnoses such as Nodules, Polyps and Paralysis only replicates a within-laryngology decision, and is unlikely to be useful at the triage point at which voice AI would most plausibly be deployed (e.g., in primary care, prior to specialist referral). For an ML system to be clinically actionable, its label set must be aligned to the decision being made at the deployment stage, which may be a coarser, framework-grounded grouping rather than a list of individual diagnoses. We therefore recommend that future studies state the clinical decision point that their model is intended to support.

#### 4.1.2 Vocal tasks

Most research is focused on using the vowel/*a*/ vocalization task. While there are good reasons to use vowel/*a*/, such as reducing risk of identifying the patient from just vowel vocalization (hence focusing on privacy), and independence from language and accent variation, it remains unclear whether optimal results can be obtained by this task alone. Emerging research suggests that connected speech may offer improved classification of voice disorders. Therefore, incorporating connected speech tasks alongside traditional vowel tasks is recommended. Ideally, use of standardised reading passages used in clinical practice (e.g., The Rainbow Passage, The Grandfather Passage) (69) should be used.

#### 4.1.3 Modern architectures and foundation models

Although Support Vector Machines combined with MFCC features dominate the reviewed corpus, a small but growing subset of articles has begun to apply transfer learning and pre-trained representation models. Nine of the 80 articles (≈11%), almost exclusively published in 2021 or later, used transfer learning, self-supervised pre-training, or audio foundation models. These include the Whisper encoder, self-supervised models combined with glottal features (70), a Vision Transformer evaluated alongside several CNN backbones (51), pre-trained speech embeddings used as features, AudioSet-pretrained networks transferred to voice (40), and ImageNet-pretrained CNN backbones (e.g., ResNet, EfficientNet, DenseNet, MobileNet, ConvNeXt) fine-tuned on spectrograms. The limited uptake of these methods is consistent with the data constraints noted earlier. With ≈25 samples per class for the majority of articles, training a Transformer from scratch is not feasible, and the MFCC-with-shallow-classifier pipeline has remained the path of least resistance. Fine-tuning of pre-trained audio models has matured into a standard approach for other low-resource speech tasks, and represents a promising direction for this domain that warrants more systematic exploration.

#### 4.1.4 Feature choice and model interpretability

MFCCs were used in 43 of the 80 articles, more than any other feature class. While MFCCs are a well-validated representation for automatic speech recognition, they were designed under a source-filter model that emphasises vocal-tract resonance and discards much of the source-level information (e.g., jitter, shimmer, glottal aperiodicity) that is most directly tied to laryngeal pathology. They are also lossy and non-invertible, which prevents reconstruction-based inspection of what a model has learned. Future studies may benefit from complementing MFCCs with an explicit voice quality or glottal-flow stream, or replacing handcrafted features altogether with learned representations from audio foundation models.

A related concern is the absence of interpretability analysis: none of the 80 reviewed articles reported any feature-attribution analysis for tabular pipelines or saliency analysis for spectrogram-based models. Interpretability is particularly valuable in this domain because it provides a route to detecting the speaker- and recording-level shortcuts that are difficult to rule out from accuracy alone, and because it is increasingly expected for any model intended for clinical use. We therefore recommend that future work include at least a basic interpretability analysis as part of the standard evaluation.

### 4.2 Testing

#### 4.2.1 Evaluation metrics

Most research uses different evaluation metrics, making comparison between studies arduous. For multi-class classification, we recommend the use of balanced accuracy (equivalent to average recall), as it reflects performance across all classes while accounting for class imbalance. Only two articles (71, 72) out of 80 that we reviewed used balanced accuracy.

#### 4.2.2 Train/test data separation

Most studies do not clearly specify how training and testing datasets are separated, particularly when using cross-validation. This is concerning, as failure to reinitialize models between folds—which is rarely reported—can lead to information leakage, especially in deep learning models. Moreover, only a few studies (26, 70, 73) explicitly ensure that speakers are disjointed across training and testing sets. Given that models are highly effective at recognizing speaker-specific traits, overlapping speakers between sets can inflate performance metrics and undermine the validity of the results.

#### 4.2.3 Class imbalance handling

Forty-one of the 80 articles had a major class imbalance, with the balancing strategies used ranging from random under- or over-sampling and SMOTE-style synthesis to class-weighted loss functions. These strategies are not equivalent: under-sampling discards real data and increases variance; over-sampling and synthesis can introduce interpolation artefacts and bias the calibration of probabilistic outputs; and class-weighted training preserves the data but does not correct for non-representativeness in the test set. Any rebalancing should therefore be applied only to the training partition, so that the test distribution remains the clinical one, and per-class recall should be reported alongside any aggregate accuracy.

#### 4.2.4 Validation strategies

Cross-validation alone, particularly on small datasets, can give an optimistic estimate of out-of-distribution performance even when applied carefully. Where data are limited, nested cross-validation should be used so that model selection and generalisation estimation are kept separate. Wherever a second clinical site or database is available, an additional held-out split should be used for a single, end-of-study estimate of out-of-distribution performance. Reporting both an in-distribution cross-validation result and an out-of-distribution single-shot result would substantially improve comparability across the literature.

#### 4.2.5 Cross-database testing

Cross-database evaluation remains rare in multi-class voice disorder classification. While binary studies—including our own (74) have increasingly adopted this approach, only one multi-class study among the 80 reviewed (65) reported such results. This is concerning, as evaluation confined to a single dataset may overestimate performance and limit generalizability. A common barrier is the mismatch in pathology labels across datasets. In such cases, we recommend mapping to shared macro-classes where possible. At minimum, researchers should report binary classification performance across datasets to guard against spurious findings.

### 4.3 Binary detection vs. multi-class classification

Despite growing interest in voice disorder classification, much research remains focused on binary detection models. While useful for early exploration, binary classification alone limits clinical applicability. Progress toward real-world deployment requires methods that can distinguish between multiple disorder types. We therefore urge researchers to prioritize multi-class classification and adapt existing datasets or methods accordingly to ensure clinical relevance and broader impact.

### 4.4 Implications

Even within the limited research on multi-class voice disorder classification, we found a high level of dissensus regarding the databases and classes used. It was therefore impractical to compare findings across studies and draw meaningful conclusions about research directions. In comparison to recommendations for voice as a biomarker in (1), which broadly address what type of research should be done, findings from this review suggest that guidelines are needed regarding *how* to conduct the research itself so that outcomes are clinically relevant. Based on analysis of the literature in this review and best practice principles in ML modelling/analysis and acknowledging clinical practice guidelines in diagnosis of voice disorders we have formulated a series of guidelines and recommendations presented in Table 2.

**Table 2:** Summary table of challenges in automatied voice disorder classification and recommendations.

Problems	Recommendations
Diagnostic classification framework used for labeling are not consistently reported	Report the classification system and consensus rating for diagnoses during data collection and when combining different classes, if necessary.
Heavy reliance on the vowel/*a*/ vocal task	Incorporate a greater variety of vocal tasks including connected speech. Test different vocal tasks at different stages to enhance the robustness of ML systems.
Inconsistent reporting of metrics	Consistently include accuracy metrics to facilitate cross-study comparisons.
Unclear separation of training and testing data	Ensure a clear separation between training and testing datasets, avoiding speaker overlap.
Lack of cross-database experiments	Validate ML systems on out-of-domain data to ensure generalizability and reliability.
Predominance of binary classification/detection research	Advance beyond binary classification by utilizing diagnostic classification frameworks to create macro classes or by precisely classifying the specific pathologies present in the data.

### 4.5 Limitations and future research

Our review does not provide a comprehensive outline of current engineering practices. While we highlighted trends in the features, models, and techniques used in the field, this does not serve as a guide for engineers seeking the best possible baseline model. This is due to the lack of consensus regarding data collection and disorder classification which confounds a meaningful review of ML approaches. Engineers looking to identify the most suitable model for a specific subset of diagnoses will need to consult our full data extract and identify the relevant articles, rather than relying on this manuscript alone.

Additionally, we have explicitly excluded the large body of research focused on binary classification tasks from our review. This decision introduces the risk of missing promising engineering practices that may already have been applied in the field, albeit limited to binary classification settings. We accept this trade-off because the review's purpose is to characterise where multi-class voice disorder classification (the task complexity required for clinical translation) currently stands; broadening the scope to binary studies would dilute that characterisation rather than sharpen it.

We recommend that future clinical research focus on developing a symptom-based classification system using a Delphi process — one that facilitates broad expert consultation and aims for consensus across the field to resolve this dissensus. In the interim, we urge engineering studies to explore alternative diagnostic classification frameworks and validate models across multiple databases to better support comparison with the broader literature.

## 5 Conclusions

This review showed that ML research in multi-class classification and discrimination of voice disorders remains largely confined to the laboratory, struggling to transition into clinical practice. There are considerable variations in diagnostic labels, discrepancies in data availability, and inconsistency in testing methodologies across previous studies. This lack of standardization precludes meaningful comparisons and limits the generalization of models. The absence of consensus throughout the classification method —from label selection to test set construction and performance metrics—seems to present a major barrier to clinical adoption. To overcome these challenges, future research should focus on achieving consensus in diagnostic classifications. In the absence of consensus, testing across multiple frameworks can enhance the comparability of results and improve the potential for broader applicability in clinical settings.

## Footnotes

^1^Different terms have been used in literature to refer to a diagnostic classification framework, such as classification group, multi-axis framework (4).

^2^ https://github.com/ComputationalAudioResearchLab/divr-scoping-review/blob/main/data/Scoping%20Review%20Extraction.xlsx
