# Supplementary material for: Voice disorders classification using machine learning: a scoping review
Source: Front Digit Health. 2026 Jun 8;8:1800132. doi: 10.3389/fdgth.2026.1800132 (PMC13284133; doi:10.3389/fdgth.2026.1800132)
Supplement: Supplementary file 2 [file Datasheet2.docx]

Search Concepts

Search concepts along with the terms identified for each concept. The asterisks in the terms indicate wildcards.

| **SEARCH CONCEPT** | **TERMS FOR EACH CONCEPT** |
| --- | --- |
| Voice Disorders | (Voice AND (disorder* OR problem* OR handicap)) OR “laryngeal disorder” OR dysphonia OR aphonia |
| Organic voice disorders | Organic AND (“voice disorder”* OR dysphonia*) OR  laryn* structural OR laryn*vascular OR laryn*malignan*OR laryn*congenital OR laryn*epithelial OR laryn*inflammat*OR laryn*paralysis OR laryn*palsy OR laryn*trauma OR laryn*neurologi*  Voice AND (disorder* OR problem* OR handicap) OR “laryngeal disorder” OR dysphonia OR aphonia AND (“myasthenia gravis" “peripheral neuropathy” OR “amyotrophic lateral sclerosis” OR “Parkinson disease” OR “multisystem atrophy” OR “spasmodic dysphonia” OR “dystonic tremor” OR “essential vocal tremor") |
| Muscle Tension dysphonia | “muscle tension” AND dysphonia OR “voice disorder*” OR “muscle misuse” AND dysphonia OR “voice disorder*” OR “ventricular dysphonia” |
| Functional voice disorders | (Functional OR psychogenic) AND (“voice disorder”* OR dysphonia* OR aphonia) OR “Functional- psychogenic voice disorder*” |
| Engineering Challenge | Classification *OR* Recognition *OR* Identification *OR* Diagnosis OR Detection OR Decision Support |
| Engineering Techniques | Machine Learning OR Deep Learning OR Artificial Intelligence OR Machine Intelligence OR Pattern Recognition OR Neural Network |
