# Supplementary material for: Voice disorders classification using machine learning: a scoping review
Source: Front Digit Health. 2026 Jun 8;8:1800132. doi: 10.3389/fdgth.2026.1800132 (PMC13284133; doi:10.3389/fdgth.2026.1800132)
Supplement: Supplementary file 3 [file Datasheet3.docx]

# Web of Science

“voice disorder*” OR “laryngeal disorder*” OR “vocal disorder*” OR dysphonia OR aphonia OR “organic voice disorder” OR “organic dysphonia” OR “muscle tension dysphonia” OR “muscle tension voice disorder*” OR “muscle misuse dysphonia*” OR “muscle misuse voice disorder*” OR “ventricular dysphonia” OR “nonorganic voice disorder*” OR “functional psychogenic voice disorder*” OR “psych* voice disorder*” OR “functional neurological voice disorder*” OR “Neuro* voice disorder*” (All Fields) AND Classification OR recognition OR identification OR diagnosis OR detection OR “decision support” OR "machine learning” OR “deep learning” OR “artificial intelligence” OR “machine intelligence” OR "pattern recognition" OR "neural network” (All Fields) and English (Languages)

# Scopus

( TITLE-ABS-KEY ( {voice disorder*} OR {laryngeal disorder*} OR {vocal disorder*} OR dysphonia OR aphonia OR {organic voice disorder} OR {organic dysphonia} OR {muscle tension dysphonia} OR {muscle tension voice disorder*} OR {muscle misuse dysphonia*} OR {muscle misuse voice disorder*} OR {ventricular dysphonia} OR {nonorganic voice disorder*} OR {functional psychogenic voice disorder*} OR {psych* voice disorder*} OR {functional neurological voice disorder*} OR {Neuro* voice disorder*} ) AND TITLE-ABS-KEY ( classification OR recognition OR identification OR diagnosis OR detection OR {decision support} OR {machine learning} OR {deep learning} OR {artificial intelligence} OR {machine intelligence} OR {pattern recognition} OR {neural network} ) ) AND PUBYEAR > 2012 AND ( LIMIT-TO ( LANGUAGE , "English" )

# Compendex

(((( (voice disorder*) OR (laryngeal disorder*) OR (vocal disorder*) OR dysphonia OR aphonia OR (organic voice disorder) OR (organic dysphonia) OR (muscle tension dysphonia) OR (muscle tension voice disorder*) OR (muscle misuse dysphonia*) OR (muscle misuse voice disorder*) OR (ventricular dysphonia) OR (nonorganic voice disorder*) OR (functional psychogenic voice disorder*) OR (psych* voice disorder*) OR (functional neurological voice disorder*) OR (Neuro* voice disorder*) )) WN All fields) AND ((( classification OR recognition OR identification OR diagnosis OR detection OR (decision support) OR (machine learning) OR (deep learning) OR (artificial intelligence) OR (machine intelligence) OR (pattern recognition) OR (neural network) )) WN All fields)), English only

# CINAHL

S45: S21 AND S42 Limiters - Published Date: 20130101-20250519 Narrow by Language: - english
S44: S21 AND S42 Limiters - Published Date: 20130101-20250519
S43: S21 AND S42
S42: S22 OR S23 OR S24 OR S25 OR S26 OR S27 OR S28 OR S29 OR S30 OR S31 OR S32 OR S33 OR S34 OR S35 OR S36 OR S37 OR S38 OR S39 OR S40 OR S41
S41: "neural network"
S40: (MH "Neural Networks (Computer)")
S39: "pattern recognition"
S38: "Artificial Intelligence"
S37: "machine intelligence"
S36: (MH "Artificial Intelligence+")
S35: "deep learning"
S34: (MH "Deep Learning")
S33: "machine learning"
S32: (MH "Machine Learning+")
S31: (MH "Decision Support Techniques+")
S30: "decision support"
S29: "detection"
S28: "Diagnosis"
S27: (MH "Diagnosis+")
S26: "identification"
S25: (MH "Voice Recognition Systems")
S24: "recognition"
S23: "Classification*"
S22: (MH "Classification+")
S21: S1 OR S2 OR S3 OR S4 OR S5 OR S6 OR S7 OR S8 OR S9 OR S10 OR S11 OR S12 OR S13 OR S14 OR S15 OR S16 OR S17
S20: "neuro* voice disorder*"
S19: "functional neurological voice disorder*"
S18: "psych* voice disorder*"
S17: "functional psychogenic voice disorder*"
S16: "nonorganic voice disorder*"
S15: "ventricular dysphonia"
S14: "muscle misuse voice disorder*"
S13: "muscle misuse dysphonia*"
S12: "muscle tension voice disorder*"
S11: "muscle tension dysphonia"
S10: (MH "Dysphonia, Muscle Tension")
S9: "organic dysphonia"
S8: "organic voice disorder"
S7: "Aphonia"
S6: (MH "Aphonia")
S5: "dysphonia"
S4: "vocal disorder*"
S3: "laryngeal disorder*"
S2: "voice disorder*"
S1: (MH "Voice Disorders+")

# Medline

1. Voice Disorders/
2. "voice disorder*".mp.
3. "laryngeal disorder*".mp.
4. "vocal disorder *".mp.
5. Dysphonia/
6. "dysphonia".mp.
7. Aphonia/
8. "Aphonia".mp.
9. "organic voice disorder".mp.
10. "organic dysphonia".mp.
11. "muscle tension dysphonia".mp.
12. "muscle tension voice disorder*".mp.
13. "muscle misuse dysphonia*".mp.
14. "muscle misuse voice disorder*".mp.
15. "ventricular dysphonia".mp.
16. "nonorganic voice disorder*".mp.
17. "functional psychogenic voice disorder*".mp.
18. "psych* voice disorder*".mp.
19. "functional neurological voice disorder*".mp.
20. "neuro* voice disorder*".mp.
21. 1 or 2 or 3 or 4 or 5 or 6 or 7 or 8 or 9 or 10 or 11 or 12 or 13 or 14 or 15 or 16 or 17 or 18 or 19 or 20
22. Classification/
23. "Classification*".mp.
24. "recognition".mp.
25. "Voice Recognition Systems".mp.
26. "identification".mp.
27. Diagnosis/
28. "Diagnosis".mp.
29. "detection".mp.
30. "decision support".mp.
31. Decision Support Techniques/
32. Diagnosis, Computer-Assisted/
33. Decision Making, Computer-Assisted/
34. Machine Learning/
35. "Machine Learning".mp.
36. Deep Learning/
37. "Deep Learning".mp.
38. Artificial Intelligence/
39. "Artificial Intelligence".mp.
40. "machine intelligence".mp.
41. Pattern Recognition, Automated/
42. "pattern recognition".mp.
43. Neural Networks, Computer/
44. Algorithms/
45. "neural network".mp.
46. 22 or 23 or 24 or 25 or 26 or 27 or 28 or 29 or 30 or 31 or 32 or 33 or 34 or 35 or 36 or 37 or 38 or 39 or 40 or 41 or 42 or 43 or 44 or 45
47. 21 and 46
48. limit 47 to english
49. limit 48 to yr="2013 - 2025"

# IEEE Explore

(
 "Full Text .AND. Metadata":"voice disorder"
 OR "Full Text .AND. Metadata":"voice disorders"
 OR "Full Text .AND. Metadata":"laryngeal disorder"
 OR "Full Text .AND. Metadata":"laryngeal disorders"
 OR "Full Text .AND. Metadata":"vocal disorder"
 OR "Full Text .AND. Metadata":"vocal disorders"
 OR "Full Text .AND. Metadata":dysphonia
 OR "Full Text .AND. Metadata":aphonia
 OR "Full Text .AND. Metadata":"organic voice disorder"
 OR "Full Text .AND. Metadata":"organic dysphonia"
 OR "Full Text .AND. Metadata":"muscle tension dysphonia"
 OR "Full Text .AND. Metadata":"muscle tension voice disorder"
 OR "Full Text .AND. Metadata":"muscle tension voice disorders"
 OR "Full Text .AND. Metadata":"muscle misuse dysphonia"
 OR "Full Text .AND. Metadata":"muscle misuse voice disorder"
 OR "Full Text .AND. Metadata":"muscle misuse voice disorders"
 OR "Full Text .AND. Metadata":"ventricular dysphonia"
 OR "Full Text .AND. Metadata":"nonorganic voice disorder"
 OR "Full Text .AND. Metadata":"nonorganic voice disorders"
 OR "Full Text .AND. Metadata":"functional psychogenic voice disorder"
 OR "Full Text .AND. Metadata":"functional psychogenic voice disorders"
 OR "Full Text .AND. Metadata":"psychological voice disorder"
 OR "Full Text .AND. Metadata":"psychological voice disorders"
 OR "Full Text .AND. Metadata":"psychogenic voice disorder"
 OR "Full Text .AND. Metadata":"psychogenic voice disorders"
 OR "Full Text .AND. Metadata":"functional neurological voice disorder"
 OR "Full Text .AND. Metadata":"functional neurological voice disorders"
 OR "Full Text .AND. Metadata":"Neurological voice disorder"
 OR "Full Text .AND. Metadata":"Neurological voice disorders"
 OR "Full Text .AND. Metadata":"Neurogenic voice disorder"
 OR "Full Text .AND. Metadata":"Neurogenic voice disorders"
 )
AND
(
 "Full Text .AND. Metadata":classification
 OR "Full Text .AND. Metadata":recognition
 OR "Full Text .AND. Metadata":identification
 OR "Full Text .AND. Metadata":diagnosis
 OR "Full Text .AND. Metadata":detection
 OR "Full Text .AND. Metadata":"decision support"
 OR "Full Text .AND. Metadata":"machine learning"
 OR "Full Text .AND. Metadata":"deep learning"
 OR "Full Text .AND. Metadata":"artificial intelligence"
 OR "Full Text .AND. Metadata":"machine intelligence"
 OR "Full Text .AND. Metadata":"pattern recognition"
 OR "Full Text .AND. Metadata":"neural network"
)

# Embase

1. "voice disorder*".mp. or voice disorder/
2. limit 1 to "remove medline records"
3. "laryngeal disorder*".mp.
4. limit 3 to "remove medline records"
5. "vocal disorder*".mp.
6. limit 5 to "remove medline records"
7. "dysphonia".mp. or dysphonia/
8. limit 7 to "remove medline records"
9. "Aphonia".mp. or aphonia/
10. limit 9 to "remove medline records"
11. "organic voice disorder".mp.
12. limit 11 to "remove medline records"
13. "organic dysphonia".mp.
14. limit 13 to "remove medline records"
15. "muscle tension dysphonia".mp.
16. limit 15 to "remove medline records"
17. "muscle tension voice disorder*".mp.
18. limit 17 to "remove medline records"
19. "muscle misuse dysphonia*".mp.
20. limit 19 to "remove medline records"
21. "muscle misuse voice disorder*".mp.
22. limit 21 to "remove medline records"
23. "ventricular dysphonia".mp.
24. limit 23 to "remove medline records"
25. "nonorganic voice disorder*".mp.
26. limit 25 to "remove medline records"
27. "functional psychogenic voice disorder*".mp.
28. limit 27 to "remove medline records"
29. "psych* voice disorder*".mp.
30. limit 29 to "remove medline records"
31. "functional neurological voice disorder*".mp.
32. limit 31 to "remove medline records"
33. "neuro* voice disorder*".mp.
34. limit 33 to "remove medline records"
35. 1 or 2 or 3 or 4 or 5 or 6 or 7 or 8 or 9 or 10 or 11 or 12 or 13 or 14 or 15 or 16 or 17 or 18 or 19 or 20 or 21 or 22 or 23 or 24 or 25 or 26 or 27 or 28 or 29 or 30 or 31 or 32 or 33 or 34
36. classification algorithm/ or "Classification*".mp.
37. limit 36 to "remove medline records"
38. "recognition".mp. or voice recognition/
39. limit 38 to "remove medline records"
40. "identification".mp.
41. limit 40 to "remove medline records"
42. "Diagnosis".mp. or computer assisted diagnosis/
43. limit 42 to "remove medline records"
44. detection algorithm/ or "detection".mp.
45. limit 44 to "remove medline records"
46. "decision support".mp. or decision support system/
47. limit 46 to "remove medline records"
48. "Machine Learning".mp. or machine learning/ or expert system/ or algorithm/ or artificial intelligence/
49. limit 48 to "remove medline records"
50. "Deep Learning".mp. or deep learning/
51. limit 50 to "remove medline records"
52. "Artificial Intelligence".mp.
53. limit 52 to "remove medline records"
54. "machine intelligence".mp.
55. limit 54 to "remove medline records"
56. "pattern recognition".mp. or pattern recognition/
57. limit 56 to "remove medline records"
58. artificial neural network/ or "neural network".mp. or convolutional neural network/
59. limit 58 to "remove medline records"
60. 37 or 39 or 41 or 43 or 45 or 47 or 49 or 51 or 53 or 55 or 57 or 59
61. 35 and 60
62. limit 61 to english language
63. limit 62 to yr="2013 - 2025"
